# Combined immunization using DNA-Sm14 and DNA-Hsp65 increases CD8+ memory T cells, reduces chronic pathology and decreases egg viability during *Schistosoma mansoni* infection

**DOI:** 10.1186/1471-2334-14-263

**Published:** 2014-05-16

**Authors:** Milena Sobral Espíndola, Fabiani Gai Frantz, Luana Silva Soares, Ana Paula Masson, Cristiane Tefé-Silva, Claudia Silva Bitencourt, Sérgio Costa Oliveira, Vanderlei Rodrigues, Simone Gusmão Ramos, Célio Lopes Silva, Lúcia Helena Faccioli

**Affiliations:** 1 Departamento de Análises Clínicas, Toxicológicas e Bromatológicas, Faculdade de Ciências Farmacêuticas de Ribeirão Preto, Universidade de São Paulo, Av. do Café s/n 14040-903 Ribeirão Preto, São Paulo, Brazil; 2 Departamento de Bioquímica e Imunologia, Faculdade de Medicina de Ribeirão Preto, Universidade de São Paulo, Av. Bandeirantes 3900, 14049-900 Ribeirão Preto, São Paulo, Brazil; 3 Departamento de Patologia, Faculdade de Medicina de Ribeirão Preto, Universidade de São Paulo, Av. Bandeirantes, 3900, 14049-900 Ribeirão Preto, SP, Brazil; 4 Departamento de Bioquímica e Imunologia, Instituto de Ciências Biológicas, Universidade Federal de Minas Gerais, Av. Antonio Carlos 6627, Pampulha, Belo Horizonte 31270-901, MG, Brazil

**Keywords:** Schistosomiasis, DNA vaccines, DNA-Hsp65, DNA-Sm14

## Abstract

**Background:**

Schistosomiasis is one of the most important neglected diseases found in developing countries and affects 249 million people worldwide. The development of an efficient vaccination strategy is essential for the control of this disease. Previous work showed partial protection induced by DNA-Sm14 against *Schistosoma mansoni* infection, whereas DNA-Hsp65 showed immunostimulatory properties against infectious diseases, autoimmune diseases, cancer and antifibrotic properties in an egg-induced granuloma model.

**Methods:**

C57BL/6 mice received 4 doses of DNA-Sm14 (100 μg/dose) and DNA-Hsp65 (100 μg/dose), simultaneously administrated, or DNA-Sm14 alone, once a week, during four weeks. Three groups were included: 1- Control (no immunization); 2- DNA-Sm14; 3- DNA-Sm14/DNA-Hsp65. Two weeks following last immunization, animals were challenged subcutaneously with 30 cercariae. Fifteen, 48 and 69 days after infection splenocytes were collected to evaluate the number of CD8+ memory T cells (CD44^high^CD62^low^) using flow cytometry. Forty-eight days after challenge adult worms were collected by portal veins perfusion and intestines were collected to analyze the intestinal egg viability. Histological, immunohistochemical and soluble quantification of collagen and α-SMA accumulation were performed on the liver.

**Results:**

In the current work, we tested a new vaccination strategy using DNA-Sm14 with DNA-Hsp65 to potentiate the protection against schistosomiasis. Combined vaccination increased the number of CD8+ memory T cells and decreased egg viability on the intestinal wall of infected mice. In addition, simultaneous vaccination with DNA-Sm14/DNA-Hsp65 reduced collagen and α-SMA accumulation during the chronic phase of granuloma formation.

**Conclusion:**

Simultaneous vaccination with DNA-Sm14/DNA-Hsp65 showed an immunostimulatory potential and antifibrotic property that is associated with the reduction of tissue damage on *Schistosoma mansoni* experimental infection.

## Background

Schistosomiasis is one of the most important neglected diseases found in developing countries and constitutes a serious public health problem that is directly related to poverty and social disadvantage [1,2]. This disease affects 249 million people worldwide [3]. The drugs that are currently available do not prevent reinfections and are inefficient against the immature stages of the worm. Therefore, the development of an efficient vaccination strategy is an essential component in the control of the disease [4,5].

Protection as a result of immunization with recombinant Sm14, a fatty acid-binding protein, ranged from 25% to 67% in mice using different approaches [6-9]. The use of DNA as a vaccine that stimulates all arms of adaptive immunity, including both cellular and humoral responses, is one of the most promising techniques for immunization against various pathogens and tumors [10]. DNA vaccines against *S. mansoni* were experimentally described against different epitopes such as Sm-p80 [11,12], Sm23 [13], SmCT-SOD [14] and a multivalent vaccine [15]. In an attempt to enhance rSm14 protection, the use of the DNA-Sm14 vaccine co-administered with the plasmid containing the gene for IL-12 altered the immunologic profile but failed to increase the protection provided by DNA-Sm14 alone, which was 40% [16]. Given the immunostimulatory and protective potential of DNA-Sm14 in the prevention against schistosomiasis, new strategies that complement and modulate the immune response generated by DNA-Sm14 are important to achieve satisfactory protection levels.

Our group has previously reported the immunostimulatory properties of the *Mycobacterium leprae* 65-kDa heat-shock protein (DNA-Hsp65), which is protective against *M. tuberculosis*[17-19] and effective as an immunomodulatory agent in several diseases, such as leishmaniasis [20], paracoccidioidomycosis [21], chromoblastomycosis [22], diabetes [23], arthritis [24], allergy [25] and phase I cancer trials [26,27]. Our group also reported that in a model of *S. mansoni* egg-induced fibrosis, the administration of DNA-Hsp65 reduced fibrotic processes during granuloma formation and led to a decrease in collagen deposition at the injury site [28]. Thus, in the current study, we tested a new vaccination strategy using DNA-Sm14 and DNA-Hsp65, where DNA-Sm14 would induce specific immune responses against *S. mansoni* and DNA-Hsp65 would potentiate the immune response in a non-specific manner. This immunization strategy would achieve optimal levels of protection and tissue preservation against *S. mansoni* infection and overcome the difficulties found in DNA-Sm14 vaccination.

## Methods

### Animals

C57BL6/6 mice (20-25 g) were obtained from the animal facilities of Faculdade de Ciências Farmacêuticas de Ribeirão Preto, Universidade de São Paulo (FCFRP - USP). All of the experiments were approved and conducted in accordance with the guidelines of the Animal Care Committee of the University (Protocol No. 09.1.144.53.3).

### Parasites and experimental infection

*Schistosoma mansoni* LE strain was maintained by routine passage through *Biomphalaria glabrata* snails and BALB/c mice (20-25 g) from the animal facilities of Faculdade de Medicina de Ribeirão Preto, Universidade de São Paulo (FMRP - USP). The infected snails were induced to shed cercariae under light exposure in water for 2 hours. The number of cercariae in suspension was determined, and the mice were subcutaneously injected with 30 cercariae/mouse with the help of a sterile syringe and a 22G1 needle (BD Biosciences).

### DNA vaccines

The Sm14 gene was isolated from the pMAL-c2/Sm14 construct by digestion with the enzymes *Xba* I and *Sal* I and subcloned into the mammalian expression vector pCI (Promega Corp., Madison, WI, USA). The pCI/Sm14 plasmid was used to transform *Escherichia coli* DH5α cells, and the clones containing the insert were selected by resistance to ampicillin. The presence of the Sm14 insert in the pCI/Sm14 construct was confirmed by restriction analysis and sequencing as previously described [16]. The Hsp65 gene was isolated from *M. leprae* and cloned into *BamH* I – *Not* I restriction sites of the pVAX1 vector (Invitrogen Corp., Carlsbad, CA, EUA). The pVAX1/Hsp65 plasmids were replicated in DH5α *Escherichia coli* cells, and the clones containing the insert were selected by resistance to kanamycin. The presence of the Hsp65 insert in the pVAX/Hsp65 construct was confirmed by restriction analysis and sequencing [17]. The pCI/Sm14 and pVAX/Hsp54 plasmids were purified using the Endofree Plasmid Giga kit (Qiagen, Valencia, CA, USA) according to the manufacturer’s protocol. The endotoxin levels were determined using a QCL-1000 Limulus amebocyte lysate kit (Cambrex Company, Walkersville, MD, USA) and were less than 0.1 endotoxin units (EU)/μg, as recommended by European and US Pharmacopoeias. pCI/Sm14 is referred here as DNA-Sm14 and pVAX/Hsp65 is referred to as DNA-Hsp65.

### Immunization procedures

C57BL/6 mice received four doses of 100 μg/each of purified DNA-Sm14 or DNA-Hsp65 in the quadriceps muscle with an interval of one week between each dose [15], on days 1, 7, 14 and 21 (100 μg in 100 μL of 50% sterile buffered sucrose). For the simultaneous administration of DNA-Sm14/DNA-Hsp65, mice received four doses of each plasmid at a final concentration of 2 μg/μL (100 μg in 50 μL of 50% sterile buffered sucrose) and administered in different legs. Thirty-six days after the beginning of immunization, the mice were infected with 30 cercariae of *S. mansoni* subcutaneously, injected using a syringe. After 15, 48 or 69 days post infection, the mice were euthanatized, and the results were analyzed. A total of eighteen animals per group were used, and each experiment was performed twice. For each time point the number of euthanatized mice was different, as following: on day 15 after infection, 3 mice per group; on day 48, 8 mice per group and on day 69, 7 mice per group were euthanatized. For each different parameter the number of mice is specified in the subtitle of the figure.

### Parasitic burden

Forty-eight days post-infection, the adult worms were recovered by hepatic and mesenteric perfusion using previously reported techniques [29] with minor modifications. The worms were counted under a dissecting microscope to evaluate the worm burden. The protection level was calculated by comparing the number of worms recovered from each experimental group and the controls using the following formula: *P* = [(*C* − *V*)/*C*] × 100, where *P* is the % protection, *C* is the mean worm count in the control animals and *V* is the mean worm count in the vaccinated animals. For parasitic burden evaluation, two independent experiments were performed, on which 6-7 animals per group were used on Experiment 1, and 7-8 animals per group on Experiment 2.

### Intestinal egg viability

Following perfusion to recover the parasites, fragments of the intestine (terminal ileum) from each animal were separated and washed in saline solution. The intestines were opened lengthwise, and the excess mucus was removed. One-centimeter fragments were cut off, partially dried on absorbent paper and placed between a glass slide and coverslip. The preparation was inverted and pressed on a rubber surface padded with filter paper [30]. The fragments were examined with an optical microscope (100×), and 200 eggs/mouse were counted and classified according to the developmental stage as follows: *(i)* viable immature eggs (1^st^ to 4^th^ stage), *(ii)* viable mature eggs or *(iii)* dead eggs [31]. The percentage of eggs in each egg stage was calculated.

### Immunophenotyping of cells by flow cytometry

After immunization and infection, the spleen memory T lymphocyte phenotypes were evaluated using flow cytometry. CD4, CD8, CD44 and CD62L expression were determined by immunostaining with antibodies conjugated to different fluorochomes (BD Biosciences, NJ, USA). Specific rat IgG2a isotype controls were used to monitor non-specific binding. The stained cells were washed with FACS buffer (PBS containing 2% fetal bovine serum (FBS) and 1 g/L of NaN_3_), pelleted by centrifugation at 400x *g* and fixed with PBS containing 1% (w/v) paraformaldehyde. A total of 30,000 events were acquired (FACSCanto TM; Becton Dickinson, CA, USA) using the FACS Diva for data acquisition and analysis. The data were analyzed using the number of events of each cell marker. Thirty thousand events per sample were collected, each sample corresponded to an individual mouse, and three-color fluorescence-activated cell analysis was performed. CD8+ CD44^hi^CD62L^low^ cells were analyzed through the expression of CD62L from CD44^hi^ CD8+ cells gated lymphocyte populations.

### Histologic and immunohistochemical analysis

Liver fragments were collected, fixed in 10% formalin and processed for paraffin embedding for the histological and immunohistochemical studies. Five-micrometer-thick sections were cut and stained with HE for histopathological analysis and Picrosirius red to visualize collagen around the granulomas. Immunohistochemical staining was performed to analyze the fibrotic marker α-SMA (Abcam). A minimum of 25 granulomas per group were measured for the analysis. The surface density of the collagen and α-SMA fibers in the liver was determined by optical density image analysis. The images were captured using a video camera (Leica® Microsystems, Heebrugg, Switzerland) coupled to a DMR microscope (Leica®, Microsystems GmbH, Wetzlar, Germany) and computer. The images were processed using Leica QWin software (Leica Microsystems Image Solutions®, Cambridge, UK). The thresholds for the collagen fibers were established for each slide after enhancing the contrast until the fibers were easily identified as red bands. The values are expressed as a percentage of the area.

### Collagen assay

Liver slices from infected mice were homogenized in a specific solution (EDTA 1 mM, Indometacin 10 μM and Protease Inhibitor Cocktail Tablets - 1 tablet/50 mL of solution). For each 100 mg of tissue, 1 mL of solution was used and homogenates were obtained after 30 seconds of homogenization, followed for 30 min centrifugation (1,200x *g*) and the total volume of supernatants were collected, which were used for soluble collagen quantification as described. 1 mL of Sircol-dye was added to 100 μL of supernatant, and the contents of the tubes were homogenized for 30 min and centrifuged for 10 min (10,000x *g*). The pellets were dissolved in 250 μL of an alkaline reagent. The absorbance was read at 540 nm. The total soluble collagen was determined using a standard curve [32] and normalized to the total protein levels (in milligrams) present in the supernatants of each sample measured by the Bradford assay as previously described [33].

### Statistical analysis

The data are represented as the mean ± SEM and were analyzed using GraphPad Prism version 5.0 (GraphPad Software, San Diego, CA). The comparisons were performed using a one-way analysis of variance with the Newman-Keuls post-test. Differences were considered significant if the *P* value was < 0.05. The experiments were repeated 2 times with similar results and for each specific assay we used a representative number of mice that is specified on the referred subtitle.

## Results

**Figure 1:**
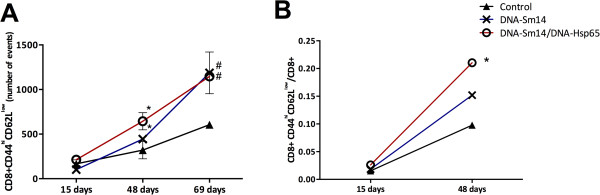
**A combined vaccination strategy using DNA-Sm14/DNA-Hsp65 increased the number of CD8+ memory T cells in the spleen of infected mice. A)** The number of CD8+ CD44^hi^CD62L^low^ memory T lymphocytes in the spleen of mice without immunization (Control) or immunized with DNA-Sm14 or DNA-Sm14/DNA-Hsp65 and infected with *S. mansoni* over time (15, 48 and 105 days after infection). **B)** The ratio of CD8+ memory T lymphocytes / total number of CD8+ in the spleen of mice vaccinated with DNA-Sm14 or DNA-Sm14/DNA-Hsp65 and infected with *S. mansoni* on days 15 and 48 post infection. After immunization, the mice were infected with 30 cercariae, and the phenotype of the memory T lymphocytes in the spleen was evaluated using flow cytometry. The results are expressed as the mean ± standard error from 3-5 animals/group and are representative of two experiments. **A)** *p < 0.05 DNA-Sm14/DNA-Hsp65 48 days vs 15 days; DNA-Sm14 48 days vs 15 days; #p < 0.05 DNA-Sm14/DNA-Hsp65 69 days vs 48 days; DNA-Sm14 69 days vs 48 days. **B)** *p < 0.05 DNA-Sm14/DNA-Hsp65 48 days vs Control group 48 days.

The main feature of vaccination is the induction of immunogenicity, in which the generation of memory cells is highly associated with protective immunity against specific antigens for long periods of time. To determine the impact of DNA-Sm14 and DNA-Sm14/DNA-Hsp65 on the generation of memory T cells, we assessed the frequencies of CD4+ and CD8+ memory T lymphocytes in the spleen of mice infected with *S. mansoni*. Vaccination with DNA-Sm14 or DNA-Sm14/DNA-Hsp65 increased the number of CD8+ CD44^hi^CD62L^low^ cells 48 and 69 days post infection (Figure 1A). The number of CD4+ CD44^hi^CD62L^low^ cells increased slightly post combined vaccination (data not shown). Moreover, we observed that immunization with DNA-Sm14/DNA-Hsp65 induced the highest ratio of memory CD8+ lymphocytes to the total number of CD8+ cells in the spleen (Figure 1B). However, the ratio of CD4+ cells did not change (data not shown). Thus, these results indicate that the combined vaccination induces the differentiation of CD8+ memory T cells but not CD4+ T cells.

**Figure 2:**
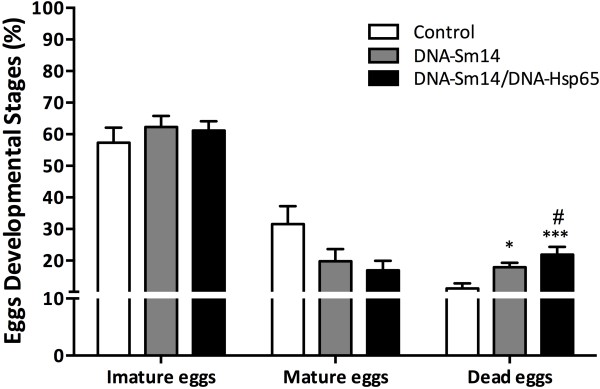
**Simultaneous vaccination with DNA-Sm14/DNA-Hsp65 reduces egg viability.** Two weeks after the last immunization, the mice were infected with 30 cercariae, and the developmental stages of the eggs were evaluated in the intestinal tissue 48 days post infection. The intestinal eggs were identified as immature, mature or dead using the Oogram technique. The results are expressed as the mean ± standard error from 6-7 animals/group and are representative of two experiments. *p < 0.05 vs Control group.

The major parameter to be considered in vaccine effectiveness is directly related to reducing the number of microorganisms in the host. Thus, we analyzed the effects of our vaccines on the reduction of parasite burden and egg viability. Vaccination with DNA-Sm14 alone induced 34.5% and 28.2% of worm reduction in the first and second sets of experiments, respectively. Simultaneous vaccination with DNA-Sm14/DNA-Hsp65 failed to reduce the worm burden in both experiments (Table 1), that could be explained by the less potent Th1 differentiation in this group observed by enhanced IgG1/IgG2a ratio (Additional file 1: Figure S1 and Table S1) compared to DNA-Sm14 and by the decreased IFN-γ release on bronchoalveolar space (Additional file 1: Figure S2). Although there was no difference in parasite load, DNA-Sm14/DNA-Hsp65 increased the amount of dead eggs in the intestinal tissue by 96.5% compared to the control group, while DNA-Sm14 only increased the amount of dead eggs by 60.35% (Figure 2). Thus, the addition of DNA-Hsp65 to the vaccination strategy decreased egg viability and possibly reduced the secretion of soluble egg antigens, which are responsible for the strong granulomatous response observed in the liver and intestine of mice.

**Table 1:** **Protection level induced by immunization with DNA-Sm14 or DNA-Sm14/DNA-Hsp65 48 days post infection with *S. mansoni***

**Experiment**	**Groups**	**Worm Burden (mean)**	**± SEM**	**n**	**Protection**
1	Control	19.86	1.405	7	-
1	DNA-Sm14	13.00*	1.024	7	34.5%^a^
1	DNA-Sm14/DNA-Hsp65	16.83	0.749	6	15.3%^a^
2	Control	18.29	0.746	7	-
2	DNA-Sm14	13.13*	1.231	8	28.2%^a^
2	DNA-Sm14/DNA-Hsp65	16.00	1.291	7	12.5%^a^

*Significant reduction in worm burden recovery compared to control group in each experiment. p < 0.05.

^a^Worm burden reduction compared to Control group in each experiment.

**Figure 3:**
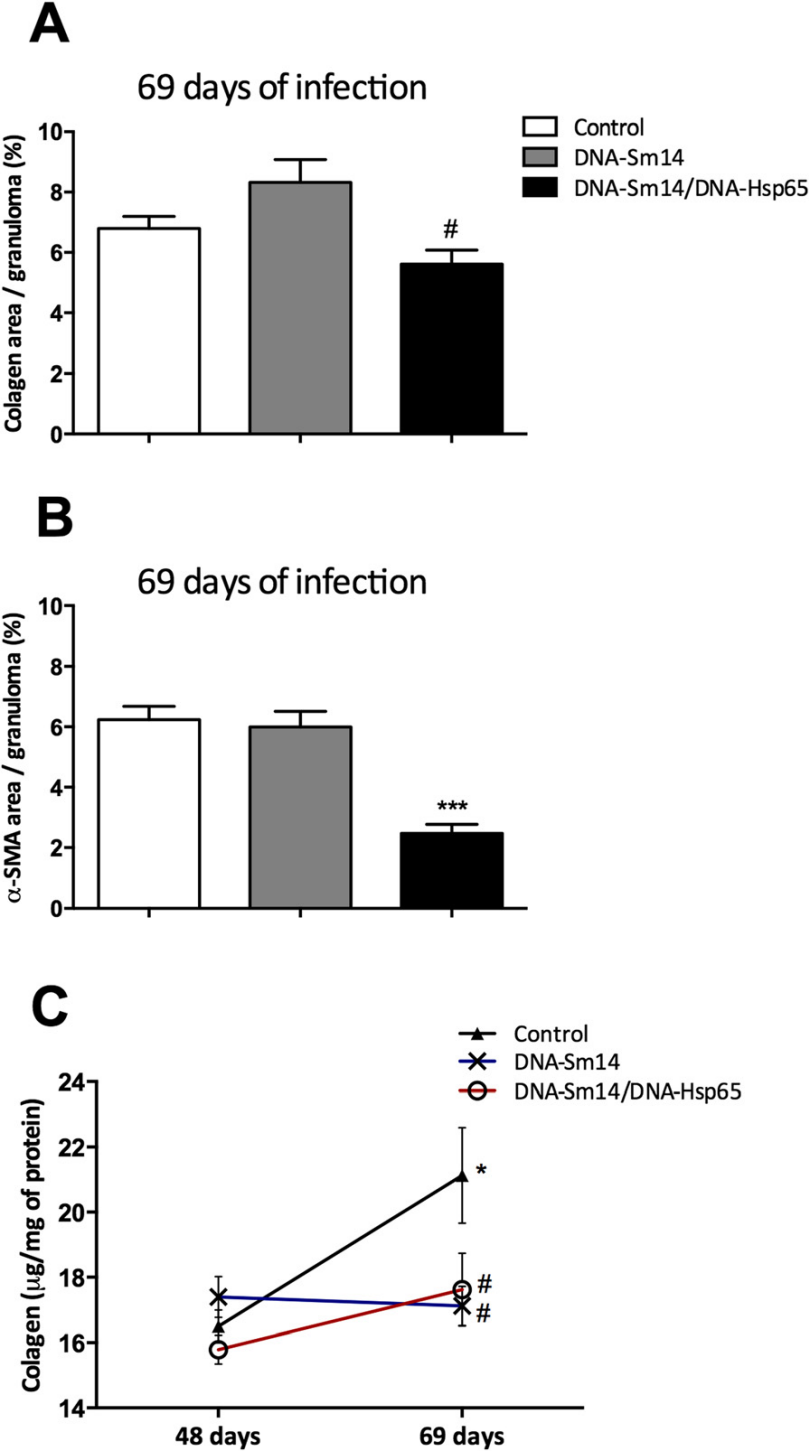
**Combined DNA-Sm14/DNA-Hsp65 vaccination reduces tissue damage during the chronic stages. A)** Collagen area and **B)** α-SMA area in liver granulomas of mice 69 days post infection with *S. mansoni* in the presence or absence of immunization with DNA-Sm14 or DNA-Sm14/DNA-Hsp65. Picrosirius staining was used to measure collagen, and immunohistochemical staining was performed to detect α-SMA. Quantitative analysis corresponding to the area occupied by collagen and α-SMA fibers were determined by digital densitometry recognition and expressed as a percentage of the total area of each granuloma. The results are expressed as the mean ± standard error of a total of 25 granulomas/group ***p < 0.001 vs Control group; #p < 0.01 vs DNA-Sm14. **C)** The concentration of acid-soluble collagen per total protein measured in the supernatants of the liver tissue homogenates. Soluble collagen was assayed using the Sircol method, and total protein was measured using the Bradford assay. The results are expressed as the mean ± standard error of 6-8 animals/group and are representative of two experiments *p < 0.05 vs Control group 48 days; #p < 0.01 vs Control group 69 days.

**Figure 4:**
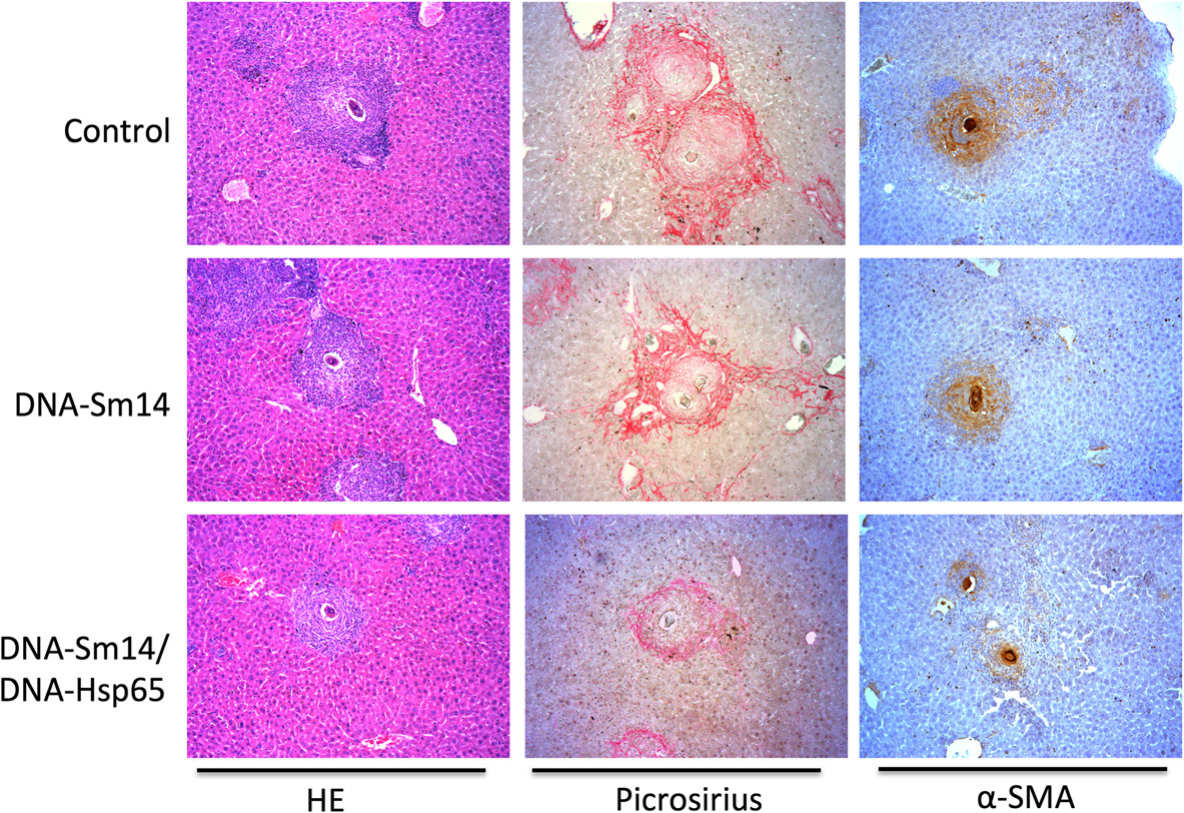
**DNA-Sm14/DNA-Hsp65 immunization reduced tissue damage during granuloma formation in the liver of *Schistosoma mansoni *infected mice.** Representative photomicrography of HE, Picrosirius and α-SMA staining from liver sections of mice without immunization (Control), immunized with DNA-Sm14 or DNA-Sm14/DNa-Hsp65, 69 days after infection. HE and Picrosirius staining were used to analyze granuloma formation and collagen deposition respectively. Immunohistochemical staining was performed to detect α-SMA fibers around the granuloma. Original magnification, X50.

A kinetic analysis of granuloma-induced fibrosis revealed that activated hepatic stellate cells (HSCs α-SMA+) and alternatively activated macrophages are the main extracellular matrix producing cells. Both cell types contribute to fibrotic tissue remodeling and collagen deposition [34]. To test whether our DNA vaccines would reduce the accumulation of extracellular matrix components, we measured α-SMA and collagen deposition around the granulomas 69 days post infection, which is considered the chronic phase, as well as the soluble collagen concentration in the liver homogenates. By day 69, DNA-Sm14/DNA-Hsp65 reduced the accumulation of collagen around the granulomas compared to immunization with DNA-Sm14 alone (Figure 3A). In addition, combined vaccination reduced the formation of α-SMA fibers (Figure 3B). The enhanced levels of soluble collagen in control group 69 days post infection were not observed on mice vaccinated with DNA-Sm14 or with both vaccines, indicating that vaccination with DNA-Sm14 alone or combined with DNA-Hsp65 decreases the extracellular matrix machinery, leading to the enhancement of tissue preservation (Figures 3C and 4).

## Discussion

The use of DNA vaccines has been widely studied as a promising tool against various diseases, including parasitic diseases. DNA vaccines not only induce potent cellular and humoral responses but also modulate the immune system by enhancing immunogenicity. Among these strategies, the use of delivery systems for DNA, including polymers, liposomes and nano- or microparticles, or even the use of adjuvants, which may be co-administered as plasmids that carry immunomodulator genes such as cytokines or costimulatory molecules, help to optimize and direct the desired response profile [35,36]. Strategies using a combination of plasmids expressing different antigens were successfully tested in human filariasis; these strategies led to an increase in immunogenicity and protection [37]. In leishmaniasis, the combination of antigens increased the number of targeted epitopes by the immune system and had an additive, perhaps even a synergistic response [38]. Therefore, we hypothesized that vaccinating with the DNA-Sm14 and DNA-Hsp65 plasmids would result in an additive modulation of the immune response.

Both vectors used in the present work were already tested on Schistosomiasis, using distinct protocols by diverse groups. pCI was used as a control on *S. mansoni* infection and did not induce protection compared to pCI/Sm14 [16]. On the other hand, pVax, without an encoding gene, was evaluated by Frantz et al., 2011, as a control in a pulmonary granuloma model, and had no effects at granuloma reduction area neither on the diminished expression of Pro-collagen1 and α-SMA after fibrosis induction. Therefore, the vectors alone had no influence on protection against *S. mansoni* experimental protocols compared to the vectors carrying the specific genes, and for these reasons were not used as a control in our work.

As the generation of memory T cells is the central event for a successful vaccination, we first investigated the frequencies of the memory T cells. Surprisingly, there was no modulation of CD4+ memory T cells with either of the vaccination strategies, which is in contrast to the idea that DNA vaccines are able to induce both subtypes of memory T cells similarly. Thus, the high frequency of memory CD8+ T cells found in mice that received DNA-Hsp65 in addition to DNA-Sm14 is in agreement with reports that Hsp65 protein and DNA preferentially induce the production of CD8+ T cells [39,40].

The addition of DNA-Hsp65 to the DNA-Sm14 strategy was not able to enhance or induce similar protection levels observed with DNA-Sm14 alone. In fact, the loss of efficacy of combined vaccination was an unexpected result, even though it has been reported that immunomodulatory molecules fail to enhance protection, as observed in a study where co-administration of DNA-Sm14 and IL-12 failed to protect against *S. mansoni* infection [16]. In our study, the higher levels of CD8+ T cell activation may explain the low efficacy of the combined vaccination protocol tested, mainly because CD8+ T cells are essential for the eradication of intracellular pathogens, while in the case of extracellular parasites, such as *S. mansoni*, these cells have no cytotoxic function.

As the major cause of death in schistosomiasis is the chronic pathology caused by egg deposition, the development of vaccines that interfere with female fecundity or egg viability is an important parameter to be evaluated. In this study, we did not observe differences in egg deposition on the intestinal wall among the groups. However, we observed that DNA-Sm14/DNA-Hsp65 enhanced the number of dead eggs compared to DNA-Sm14 alone. This reduction in egg viability is in agreement with previous studies that used Sm14 peptides for the vaccinations [41]. Thus, although the combination of vaccines was unable to protect against infection, vaccination may have played a positive role in the pathology caused by the eggs and the control of disease dissemination. In this sense, the dead eggs dispensed into the environment would be unable to continue the life cycle of the parasite.

Granuloma formation is the consequence of egg deposition in the liver and is characterized by a gradual accumulation of neutrophils, eosinophils and mononuclear cells. Tissue damage caused by profibrotic cells and components [34] are targeted by vaccines that aim to reduce injury. Previous studies have characterized DNA-Hsp65 as antifibrotic due to its ability to reduce tissue damage in a model of induced granuloma in the lung [28]. In that particular study, a static model with no interference of the parasite was used, and the studies were all performed in the lung 8 days post egg injection. Here, we used a dynamic model of infection where the effects of the vaccines were possibly modulated by immune induction mediated by the worms during the course of infection. Using different approaches, we were able to observe the potential benefits of DNA-Hsp65 in reducing chronic pathology in the liver of infected mice. The antifibrotic role was observed by a reduction of soluble and tissue collagen, α-SMA accumulation around the granulomas 69 days post infection and the maintenance of soluble collagen levels between 48 and 69 days post infection. These findings confirm the immunostimulatory characteristics of DNA-Hsp65, which are directly linked to a reduction in chronic pathology and tissue damage during schistosomiasis infection.

## Conclusion

**Figure 5:**
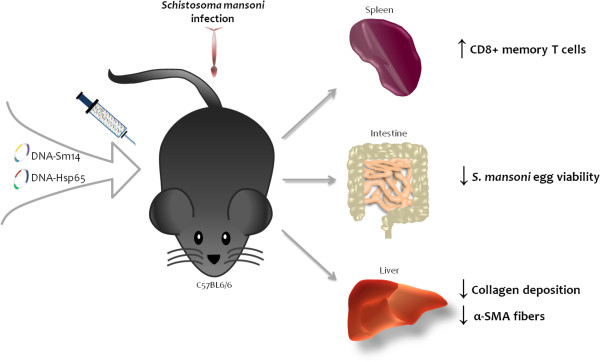
**Protective effects of DNA-Sm14/DNA-Hsp65 immunization in mice infected with** ***Schistosoma mansoni.***

Simultaneous vaccination with DNA-Sm14/DNA-Hsp65 increased the number of CD8+ memory T cells and decreased egg viability on the intestinal wall of infected mice. In addition, combined vaccination reduced collagen and α-SMA accumulation during the chronic phase of granuloma formation, indicating a potential antifibrotic property of DNA-Hsp65 that is associated with the reduction of tissue damage (Figure 5).

## Competing interests

The authors declare that they have no competing interests.

## Authors’ contributions

MSE, FGF: Conceived and designed the experiments; MSE, FGF, LSS, APM, CTS and CSB: Performed the experiments; MSE and FGF: Analyzed the data; LHF, SGR, CLS, VR, SCO: Contributed reagents/materials/analysis tools; MSE, FGF and LHF: Wrote the manuscript. All authors read and approved the final manuscript.

## Pre-publication history

The pre-publication history for this paper can be accessed here:

http://www.biomedcentral.com/1471-2334/14/263/prepub

## Supplementary Material

Additional file 1: Figure S1 Immunological profile of DNA-SM14/DNA-Hsp65 did not overlap immune induction of DNA-Sm14. **Table S1.** Anti-Sm14 IgG1/IgG2a ratio at different days after immunization with DNA-Sm14 or DNA-Sm14/DNA-Hsp65. **Figure S2.** IFN-γ concentration on bronchoalveolar lavage from mice immunized or not with DNA-Sm14, DNA-Hsp65 or DNA-Sm14/DNA-Hsp65 and infected with *S. mansoni*. Click here for file
