# Supplementary material for: Combined immunization using DNA-Sm14 and DNA-Hsp65 increases CD8+ memory T cells, reduces chronic pathology and decreases egg viability during Schistosoma mansoni infection
Source: BMC Infect Dis. 2014 May 16;14:263. doi: 10.1186/1471-2334-14-263 (PMC4031977; doi:10.1186/1471-2334-14-263)
Supplement: Additional file 1: Figure S1 — Immunological profile of DNA-SM14/DNA-Hsp65 did not overlap immune induction of DNA-Sm14. Table S1. Anti-Sm14 IgG1/IgG2a ratio at different days after immunization with DNA-Sm14 or DNA-Sm14/DNA-Hsp65. Figure S2. IFN-γ concentration on bronchoalveolar lavage from mice immunized or not with DNA-Sm14, DNA-Hsp65 or DNA-Sm14/DNA-Hsp65 and infected with S. mansoni. [file 1471-2334-14-263-S1.pdf]

Additional File 1

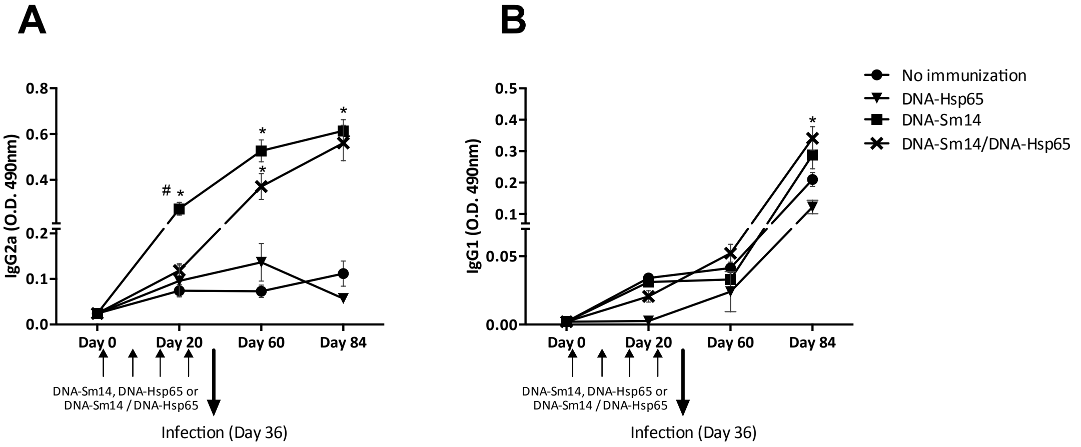

Figure S1. Immunological profile of DNA-SM14/DNA-Hsp65 did not overlap immune induction of DNA-Sm14. A and B) Specific anti-Sm14 antibody measurement after immunization with DNA-Sm14, DNA-Hsp65 or DNA-Sm14/DNA-Hsp65. **A)** IgG1 anti-Sm14**; B)** IgG2a anti-Sm14. The measurement of specific antibodies from C57BL/6 mice without immunization, immunized with DNA-Sm14 or DNA-Sm14/DNA-Hsp65 was performed 20, 60 and 84 days after the first immunization by ELISA. The infection (↓) with 30 cercariae was performed on day 36 after the beginning of vaccination. The sera were diluted 1:200 for IgG1 and 1:20 for IgG2a. The results were expressed as mean $\pm$ standard error from 5-7 animals/group. (↑) indicates the vaccination days. *p<0,05 vs mice without immunization; # p< 0,05 vs mice immunized with DNA-Sm14/DNA-Hsp65.

Table S1. Anti-Sm14 IgG1/IgG2a ratio at different days after immunization with DNA-Sm14 or DNA-Sm14/DNA-Hsp65

Days[1]	Groups	
	DNA-Sm14	DNA-Sm14/DNA-Hsp65
20	0.11	0.17
60	0.06	0.14
84	0.47	0.61

[1] days after the beginning of immunization

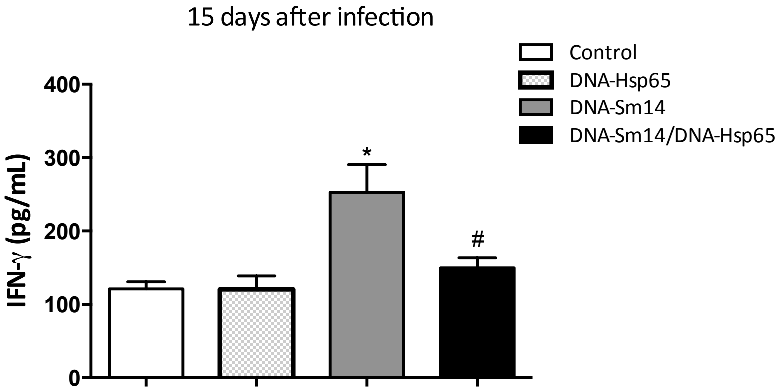

Figure S2. IFN-γ concentration on bronchoalveolar lavage from mice immunized or not with DNA-Sm14, DNA-Hsp65 or DNA-Sm14/DNA-Hsp65 and infected with *S. mansoni*. Bronchoalveolar lavage was collected 15 days after infection and INF-γ was measured by ELISA. The results were expressed as mean ± standard error from 5-7 animals/group. #p<0,05 vs DNA-Sm14.
